# Supplementary material for: An anti-collusion attack defense method for physical layer key generation scheme based on transmission delay
Source: PeerJ Comput Sci. 2023 Apr 24;9:e1349. doi: 10.7717/peerj-cs.1349 (PMC10280582; doi:10.7717/peerj-cs.1349)
Supplement: Supplemental Information 2 [file peerj-cs-09-1349-s002.pdf]

APPENDIX

Appendix A. Proof of monotonicity of $p_{success\_I}$

Lemma 1. *Let $F(n) = \sum_{r=0}^{n} C_n^r \alpha^r (1-\alpha)^{n-r} f(r)$, $0 < \alpha < 1$, if the sequence $f(n)$ is monotone increasing sequence, then the sequence $F(n)$ is monotone increasing sequence.*

Proof. Let $w(n,r) = C_n^r \alpha^r (1-\alpha)^{n-r}$. Then, we have

$$
\begin{aligned}
&F(n+1) - F(n) \\
&= \sum_{r=0}^{n+1} w(n+1,r) f(r) - \sum_{r=0}^{n} w(n,r) f(r) \\
&= \sum_{r=0}^{n+1} w(n+1,r) f(r) - (\alpha + 1 - \alpha) \sum_{r=0}^{n} w(n,r) f(r) \\
&= (1-\alpha)^{n+1} f(0) + \alpha^{n+1} f(n+1) + (1-\alpha) \sum_{r=1}^{n} w(n,r) f(r) \\
&+ \alpha \sum_{r=1}^{n} w(n,r-1) f(r) - (\alpha + 1 - \alpha) \sum_{r=0}^{n} w(n,r) f(r) \\
&= (1-\alpha)^{n+1} f(0) + \alpha^{n+1} f(n+1) - (1-\alpha)^{n+1} f(0) \\
&+ \alpha \sum_{r=0}^{n-1} w(n,r) f(r+1) - \alpha \sum_{r=0}^{n} w(n,r) f(r) \\
&= \alpha^{n+1} f(n+1) + \alpha \sum_{r=0}^{n-1} w(n,r) [f(r+1) - f(r)] - \alpha^{n+1} f(n) \\
&= \alpha \sum_{r=0}^{n} w(n,r) [f(r+1) - f(r)] > 0
\end{aligned} \tag{1}
$$

Since $[1 - (1 - \frac{r}{m})^N]^k$ is monotonically increasing with respect to r, according to Lemma. 1, we have $p_{success\_I}(n+1) > p_{success\_I}(n)$. □

Appendix B. Evidence of $p_{expose}(\alpha n) \approx p_{success\_I}(n)$

Let $w(n,r) = C_n^r \alpha^r (1-\alpha)^{n-r}$, $0 < \alpha < 1$, then, $w(n,r)$ has the following properties:

- **Property 1** $\exists r_{otp}$, $w(r_{otp}) = \max_r w(n,r)$, and $r_{otp} = [\alpha(n+1)]$, where $[x]$ represents the rounding down of x.

  Proof. Consider the following inequality:

  $$
  \begin{aligned}
  &w(n,r+1) - w(n,r) \\
  &= C_n^{r+1} \alpha^{r+1} (1-\alpha)^{n-r-1} - C_n^r \alpha^r (1-\alpha)^{n-r} \\
  &= [(\frac{n-r}{r+1})(\frac{\alpha}{1-\alpha}) - 1] w(n,r) \geq 0
  \end{aligned} \tag{2}
  $$

  Since $w(n,r) > 0$, Ineq. (2) is equivalent to

  $$(\frac{n-r}{r+1})(\frac{\alpha}{1-\alpha}) \geq 1 \tag{3}$$

  □

  The solution of Ineq. (3) is $r+1 \leq \alpha(n+1)$. So $r_{otp} = [\alpha(n+1)]$.

- **Property 2** Let $w(n, r_{otp} + \Delta r) = w(n, r_{otp}) \beta(\Delta r)$, then $0 < \beta(\Delta r) \leq 1$ and $\beta(\Delta r)$ decreases as $|\Delta r|$ increases.

Proof. Since $w(n,r) > 0$ and we have

$$\beta(\Delta r) = \frac{w(n, r_{otp} + \Delta r)}{w(n, r_{otp})} \leq \frac{w(n, r_{otp})}{w(n, r_{otp})} = 1 \tag{4}$$

so $0 < \beta(\Delta r) \leq 1$.

Besides, when $\Delta r > 0$, we have

$$\begin{aligned} \frac{\beta(\Delta r + 1)}{\beta(\Delta r)} &= \frac{w(n, r_{otp} + \Delta r + 1)}{w(n, r_{otp} + \Delta r)} \\ &< \frac{w(n, r_{otp} + \Delta r)}{w(n, r_{otp} + \Delta r)} = 1 \end{aligned} \tag{5}$$

Similarly, when $\Delta r < 0$, $\frac{\beta(\Delta r+1)}{\beta(\Delta r)} < 1$ is also true. □

Let $W(\alpha) = [w(n,0), w(n,1), \ldots, w(n,n)]$, then the distribution characteristics of $W(\alpha)$ is shown in Fig. A1.

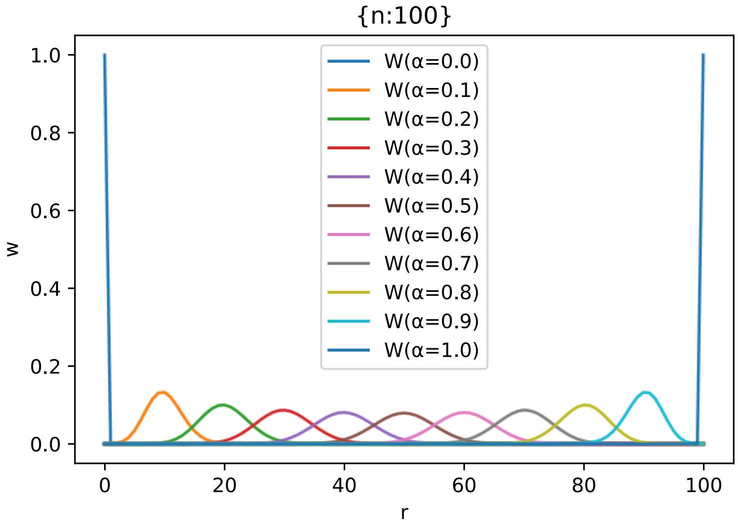

Figure A1. The distribution characteristics of $W(\alpha)$

It's clear from Fig. A1 that r_{otp} increases linearly as α increase, and $F(n)$ can be approximated by few $w(n,r)f(r)$ terms, that is

$$\begin{aligned} F(n) &= \sum_{r=0}^{n} w(n,r) f(r) \\ &\approx w(n, r_{otp}) f(r_{otp}) + \sum_{i=0}^{\Delta r} w(n, r_{otp}) \beta(\pm i) f(r_{otp} \pm i) \\ &\approx w(n, r_{otp})[1 + \sum_{i=0}^{\Delta r} \beta(\pm i)] f(r_{otp}) \\ &= \sum_{r=0}^{n} w(n,r) f(r_{otp}) = f(r_{otp}) = f(\alpha(n+1)) \end{aligned} \tag{6}$$

Therefore, we have

$$\begin{aligned} p_{success\_I}(n) &= \sum_{r=0}^{n} w(n,r) p_{expose}(r) \\ &\approx p_{expose}(\alpha(n+1)) \approx p_{expose}(\alpha n) \end{aligned} \tag{7}$$

Appendix C. An appropriate value of μ

By calculating all possible values of $p_{success\_II}$ under all possible n, the value of n_{II_otp} under the experiment can be selected as the experimental result. The experimental result is discrete, so the curve will be stepped. Then, the approximate results are calculated by Eq. (8), and by comparing them with each other we can get the value of μ.

$$n_{II\_otp} \approx < \frac{mk}{k(N-1) - mln\alpha} >, \alpha \leq \mu \tag{8}$$

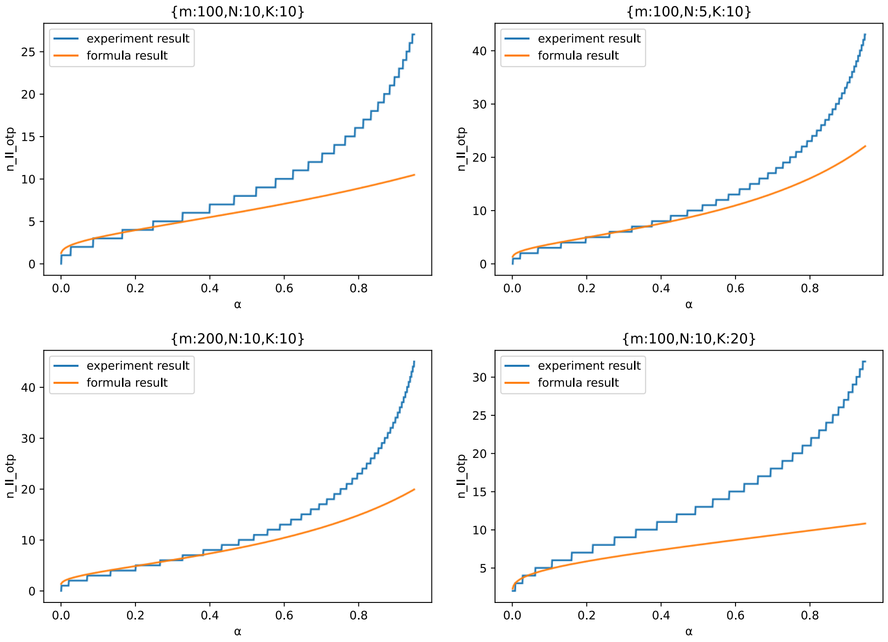

Figure A2. Comparison between approximate solution of n_{II_otp} and experimental solution

It can be seen from Fig. A2 that μ is not static but increases with the decrease of N and the increase of m, and it decreases as k increases. However, it is not significant to analyze the relationship between μ and m, N, k, so the available value of μ we choose is 0.2 according to the experimental results. Usually, in the scenario of “Cautious Defender”, $\alpha \leq 0.2$ can be satisfied, and then the attacker can calculate the best attack strategy more quickly with approximate formula.
